# Normative Scores for the NIH Toolbox Dynamic Visual Acuity Test from 3 to 85 Years

**DOI:** 10.3389/fneur.2014.00223

**Published:** 2014-10-30

**Authors:** Carol Li, Jennifer L. Beaumont, Rose Marie Rine, Jerry Slotkin, Michael C. Schubert

**Affiliations:** ^1^Department of Otolaryngology-Head and Neck Surgery, The Johns Hopkins University School of Medicine, Baltimore, MD, USA; ^2^Department of Medical Social Sciences, Feinberg School of Medicine, Northwestern University, Chicago, IL, USA; ^3^Specialty Therapy Source LLC, Jacksonville, FL, USA; ^4^Marshall University School of Medicine, Huntington, WV, USA; ^5^Department of Physical Medicine and Rehabilitation, The Johns Hopkins University School of Medicine, Baltimore, MD, USA

**Keywords:** dynamic visual acuity, gaze stability, NIH Toolbox, vestibular hypofunction, vestibular test

## Abstract

As part of the National Institutes of Health Toolbox initiative, a computerized test of dynamic visual acuity (cDVA) was developed and validated as an easy-to-administer, cost- and time-efficient test of vestibular and visual function. To establish normative reference values, 3,992 individuals, aged 3–85 years, without vestibular pathology underwent cDVA testing at multiple clinical research testing facilities across the United States. Test scores were stratified by sociodemographic characteristics. cDVA was worse in males (*p* < 0.001) and those subjects 50 years or older, while there was no difference in dynamic visual acuity across age groups binned from 3 to 49 years. Furthermore, we used these normative cDVA data as a criterion reference to compare both the long (validated) and short versions of the test. Both versions can distinguish between those with and without vestibular pathology (*p* = 0.0002 long; *p* = 0.0025 short). The intraclass correlation coefficient between long- and short-cDVA tests was 0.86.

## Introduction

The vestibulo-ocular reflex (VOR) prevents retinal slip during head motion by moving the eyes contrary to the head. This gaze stabilization occurs for both linear and angular head motion, and pathology of the VOR leads to poor visual acuity during head motion. The dynamic visual acuity (DVA) test, in which scores reflect the difference in visual acuity between stationary and head rotation, measures the individual’s ability to maintain gaze during head rotation. As part of the National Institutes of Health Toolbox for the Assessment of Neurological and Behavioral Function (NIH Toolbox) initiative (1), a computerized dynamic visual acuity (cDVA) test was developed for examination of the VOR. The NIH Toolbox cDVA test was validated in 318 individuals, aged 3–85 years. Bithermal caloric, rotational chair testing, and light box testing were completed to confirm vestibular and visual function status and shown to be reliable for static visual acuity (SVA) and DVA (2, 3).

The cDVA test has been shown to effectively evaluate the ability to see clearly during head rotation (4–6); however, many studies are limited to the adult population. While few studies have investigated the utility of the DVA test in children, the reported sensitivity (88–100%) and specificity (68–100%) is very good for identifying vestibular hypofunction (VH) (7–9). Although the evidence for implementation of the DVA test to screen for and diagnose vestibular pathology in the pediatric population is promising, the existing literature necessitates reference DVA scores derived from large, diverse populations with a wide age range.

In this study, we report the normative scoring of the NIH Toolbox cDVA from 3,992 individuals, aged 3–85 years, captured from various regions of the United States. Additionally, long and short versions of the NIH Toolbox cDVA test were compared and validated. We anticipate these data will assist the assessment of vestibular function, the interpretation of cDVA scores, and be useful for researchers interested in using the NIH Toolbox cDVA test for population-based epidemiological studies.

## Materials and Methods

### Subjects

To obtain normative values, 4,859 subjects were recruited from databases assembled through online self-enrollment, enrollment events, and random telephone calls by market research companies, Delve, La Verdad, and Facts ‘n Figures. A stratified recruitment plan by the NIH Toolbox outlining the overall norming plans for the NIH Toolbox is available (10). Individuals included community-dwelling children and adults, English and Spanish speakers, who were capable of following test instructions (English or Spanish) and able to provide informed consent or, in the case of children 8 years and older, give assent with accompanying informed consent by the parent or guardian. Individuals were excluded if they could not participate due to blindness or reading impairment. In addition to cDVA testing, sociodemographic characteristics such as age, gender, ethnicity, dominant language, and education level were collected. A total of 3,992 of the overall norming study participants completed the cDVA testing.

In a separate study, 22 subjects were recruited from an outpatient otolaryngology clinic to validate a shorter version of the NIH Toolbox cDVA test. Twelve subjects were individuals with unilateral or bilateral VH, diagnosed by caloric testing and/or quantitative head-impulse testing. Ten subjects were individuals with no history of vestibular deficits, confirmed by negative clinical head-impulse testing. All subjects gave informed consent. cDVA testing protocols were approved by the institutional review board at the Johns Hopkins University School of Medicine. Individuals were excluded if they could not participate due to blindness, oculomotor impairment, poor neck range of motion, or cervical spine instability.

### NIH Toolbox computerized dynamic visual acuity test

A cost-effective cDVA test was developed in order to minimize motor, language, and cultural influences through software written in Python and C^++^ (2). The hardware consisted of a 2-GHz Intel dual central processing unity laptop with 2 GB of RAM (IBM Thinkpad; IBM, Armonk, NY, USA), connected to a 1440 × 900 resolution monitor for optotype display. A single-axis rate sensor (O-Navi, Vista, CA, USA) attached to a soft bicycle light strap was used for detecting horizontal head rotation. Delve field technicians were trained and certified by the NIH Toolbox staff to administer the cDVA test at various Delve clinical research testing sites throughout the United States, including Atlanta, Chicago-Oak Brook, Cincinnati, Columbus, Dallas, Los Angeles, Minneapolis, Philadelphia, Phoenix, and St. Louis.

Participants were seated 12.5 ft away from the monitor at eye level. An initial SVA test was completed by presenting a single random optotype starting at size 20/80. Subjects were asked to identify five optotypes per acuity level that reduced in steps of 0.1 logarithm of the minimum angle of resolution (logMAR). This continued until either one of two conditions were met: the smallest size acuity level was reached and all five optotypes were correctly identified (20/10) or the acuity level where at least three of five optotypes were correctly identified. For the cDVA test, participants were asked to move their head side to side as if indicating “no.” When head velocity met or exceeded 180°/s, the rate sensor triggered the software to flash an optotype starting at three sizes above their predetermined SVA. The optotype remained on the screen for 83 ms and only at velocities above the threshold of 180°/s. The optotype did not flash for head velocities below this threshold. Prior to data collection, practice trials displaying multiple optotypes, size 1.30 and 1.00 logMAR, were conducted to familiarize the participant to the test and minimize learning effects. For both the SVA and DVA tests, only the letters H, O, T, and V are used for ages 3–7, while ages 8+ use the entire early treatment diabetic retinopathy study (ETDRS) letter set. In the development and validation phases of the NIH Toolbox DVA test, five letters were administered per line (optotype size) (2, 3). For this norming study, to make administration time more manageable, the number of letters administered per line was reduced from five to two and an ancillary study was conducted to compare the long (five optotype) and short (two optotype) versions of the cDVA test. Detailed test administration instructions can be found at www.nihtoolbox.org (11).

### Evaluation of post-validation changes to the NIH Toolbox cDVA test

Twelve participants with confirmed VH and 10 participants with no vestibular pathology underwent two versions of the cDVA test in the same testing session. The long version of the cDVA test displayed five letters per acuity level and the short version of the cDVA test displayed two letters per acuity level. Criteria used for stopping the short version were identical with the long version (11). Diagnostic criteria for VH included unilateral (>25% weakness) or bilateral reduced responses during bithermal caloric testing and/or horizontal VOR gain <0.7 during quantitative head-impulse testing with video-oculography (VOG). The test order, i.e., which version of the cDVA test was administered first, was randomly assigned. Eleven participants underwent the long version of the cDVA test first while the other 11 participants underwent the short version of the cDVA test first.

### Analysis

The difference between static and dynamic scores was calculated separately for leftward and rightward head rotation and represents the vestibular contribution to gaze stability. Left and right difference scores were averaged to obtain one DVA score for each individual.

Descriptive statistics including means, SD, percentiles, and ranges were calculated for each age group. The effect of age on DVA scores in the normative population was determined using regression analysis. Analysis of variance, *t*-tests, and linear regression were used to compare DVA scores among other sociodemographic categories. An alpha of *p* < 0.01 was considered statistically significant.

To validate the short version of the cDVA test (two letters per optotype size), DVA scores for subjects with and without VH, were compared using a *t*-test. Within-subject comparisons between the two test versions were performed using a paired *t*-test. *p* < 0.01 was considered statistically significant. Reliability of the shorter NIH Toolbox cDVA test was assessed using intraclass correlation coefficients (ICCs). Sensitivity, specificity, positive predictive value (PPV), and negative predictive value (NPV) were evaluated using the following equations:

$$\begin{array}{llll} \mathrm{Sensitivity} & =\frac{\mathrm{True}\text{\_}\mathrm{Positives}}{\mathrm{True}\text{\_}\mathrm{Positives}+\mathrm{False}\text{\_}\mathrm{Negatives}}\times 100\%,\; & & \;\\ \mathrm{Specificity} & =\frac{\mathrm{True}\text{\_Negatives}}{\mathrm{True}\text{\_Negatives+False\_Positives}}\times 100\%,\; & & \;\\ \mathrm{PPV} & =\frac{\mathrm{True}\text{\_Positives}}{\mathrm{True}\text{\_Positives+False\_Positives}}\times 100\%,\; & & \;\\ \mathrm{NPV} & =\frac{\mathrm{True}\text{\_Negatives}}{\mathrm{True}\text{\_Negatives+False\_Negatives}}\times 100\%.\; & & \; \end{array}$$

Criterion for abnormal scores was derived from the norming scores in the population of 4,369 participants and was calculated as the age-matched mean DVA score +2SD.

## Results

### NIH Toolbox cDVA normative data

In the normative population, 3,992 participants with cDVA data were evaluated with mean age 21.0 ± 18.9 years, range 3–85 years (Table 1). Forty-six percent were male, 50% were Non-Hispanic White, 13% were Non-Hispanic Black, and 32% were Hispanic. Thirty-four percent of the participants reported a less than high-school education level, 21% reported graduating high school or passing the GED tests, and 45% reported a higher than high-school education level. Eighty percent of participants were English-speaking, 11% were Spanish-speaking, and 9% were bilingual.

**Table 1:** **Normative population characteristics**.

	No.	Percent
**Age group**	
3	54	1.35
4	152	3.81
5	169	4.23
6	177	4.43
7	230	5.76
8	188	4.71
9	195	4.88
10	216	5.41
11	193	4.83
12	200	5.01
13	199	4.98
14	215	5.39
15	208	5.21
16	201	5.04
17	202	5.06
18–29	242	6.06
30–39	275	6.89
40–49	224	5.61
50–59	170	4.26
60–69	126	3.16
70–85	156	3.91
**Gender**	
Male	1821	45.62
Female	2171	54.38
**Ethnicity**	
White	1966	49.78
Black	531	13.45
Hispanic	1282	32.46
Other	170	4.3
**Education**	
<High school	1284	34.22
HS diploma/GED	794	21.16
>High school	1674	44.62
**Language**	
English	3183	79.73
Spanish	433	10.85
English (bilingual)	127	3.18
Spanish (bilingual)	249	6.24

**Figure 1:**
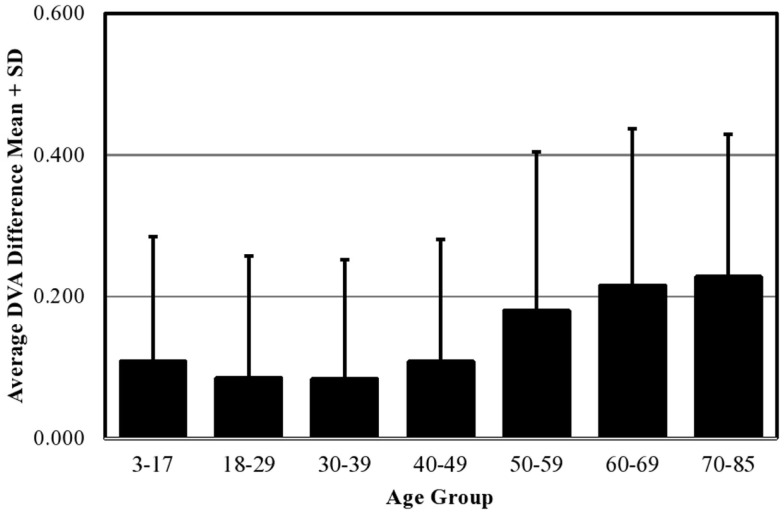
**Effect of age on mean DVA scores (mean + SD) in the normative population**.

Mean DVA score across all ages in the normative population was 0.116 ± 0.184 logMAR. Descriptive statistics including means, SD, percentiles, and ranges by age group are shown in Table 2. We combined individuals aged 3–17 years into a single age group and analyzed the effect of age as a categorical variable considering the 18–29-year group as the reference group. In multiple regression analysis adjusting for all sociodemographic characteristics, we found no difference in cDVA between those groups aged 3–49 years (Table 3). cDVA scores were significantly higher for individuals age 50 and older (*p* < 0.001) (Figure 1). Additionally, cDVA scores were higher (worse) in males (adjusted analyses *p* < 0.001). In multiple regression analysis, cDVA score did not differ among various ethnicities, education levels, or language groups (Table 3).

**Table 2:** **Normative values of the NIH Toolbox DVA test by age group**.

Participant	*N*	Mean	SD	Minimum	25th Percentile	Median	75th Percentile	Maximum
Age (years)	
3	54	0.258	0.251	−0.275	0.065	0.240	0.405	0.950
4	152	0.157	0.233	−0.250	0.015	0.115	0.240	1.260
5	169	0.105	0.174	−0.390	−0.005	0.095	0.175	1.075
6	177	0.103	0.170	−0.320	−0.015	0.100	0.195	0.730
7	230	0.085	0.166	−0.300	−0.015	0.077	0.155	0.915
8	188	0.148	0.170	−0.270	0.040	0.125	0.235	0.915
9	195	0.118	0.173	−0.335	0.000	0.090	0.215	0.990
10	216	0.109	0.159	−0.410	0.000	0.097	0.210	0.695
11	193	0.122	0.173	−0.210	0.015	0.095	0.190	0.850
12	200	0.090	0.169	−0.280	−0.025	0.070	0.175	0.945
13	199	0.091	0.174	−0.360	−0.005	0.065	0.160	1.025
14	215	0.106	0.180	−0.200	−0.015	0.080	0.195	1.265
15	208	0.095	0.141	−0.225	−0.005	0.080	0.185	0.740
16	201	0.074	0.176	−0.355	−0.035	0.055	0.145	1.170
17	202	0.090	0.169	−0.190	−0.010	0.065	0.170	1.205
18–29	242	0.084	0.173	−0.295	−0.015	0.067	0.150	1.470
30–39	275	0.084	0.169	−0.300	−0.030	0.065	0.165	0.830
40–49	224	0.108	0.173	−0.605	−0.005	0.080	0.193	0.995
50–59	170	0.180	0.225	−0.250	0.030	0.142	0.265	1.045
60–69	126	0.215	0.222	−0.310	0.080	0.177	0.300	0.970
70–85	156	0.228	0.202	−0.385	0.090	0.215	0.340	0.935
Total	3992	0.116	0.184	−0.605	0.000	0.095	0.200	1.470

**Table 3:** **Mean DVA score stratified by sociodemographic characteristics**.

	Mean DVA	SD	Unadjusted *p*	Adjusted *p*	
**Age group**				
3–17	0.108	0.177	0.497	0.137	
18–29	0.084	0.173	Reference	Reference	
30–39	0.084	0.169	0.976	0.909	
40–49	0.108	0.173	0.164	0.215	
50–59	0.180	0.225	<0.001	<0.001	
60–69	0.215	0.222	<0.001	<0.001	
70–85	0.228	0.202	<0.001	<0.001	
**Gender**				
Male	0.134	0.187	Reference	Reference	
Female	0.101	0.180	<0.001	<0.001	
**Ethnicity**				
White	0.116	0.181	Reference	Reference	
Black	0.115	0.191	0.923	0.455	
Hispanic	0.119	0.188	0.650	0.171	
Other	0.097	0.168	0.199	0.470	
**Education**				
<High school	0.114	0.181	0.990	0.426	
HS diploma/GED	0.114	0.175	Reference	Reference	
>High school	0.118	0.190	0.657	0.216	
**Language**				
English	0.115	0.182	Reference	Reference	
Spanish	0.133	0.201	0.065	0.955	
English (bilingual)	0.112	0.152	0.836	0.681	
Spanish (bilingual)	0.098	0.186	0.158	0.026	

*Unadjusted *p*-value refers to bivariate analyses. Adjusted *p*-values refer to multiple regression analysis including age, gender, ethnicity, education level, and language data*.

### Validation of the NIH Toolbox short-cDVA test

We evaluated twelve individuals (42% female, mean 51.9 ± 10.4 years) with VH, confirmed by caloric testing and/or quantitative head-impulse testing, along with ten individuals (30% female, mean 42.4 ± 17.3 years) without a history of vestibular disease. There were no significant differences in demographic characteristics between those with and without VH.

**Figure 2:**
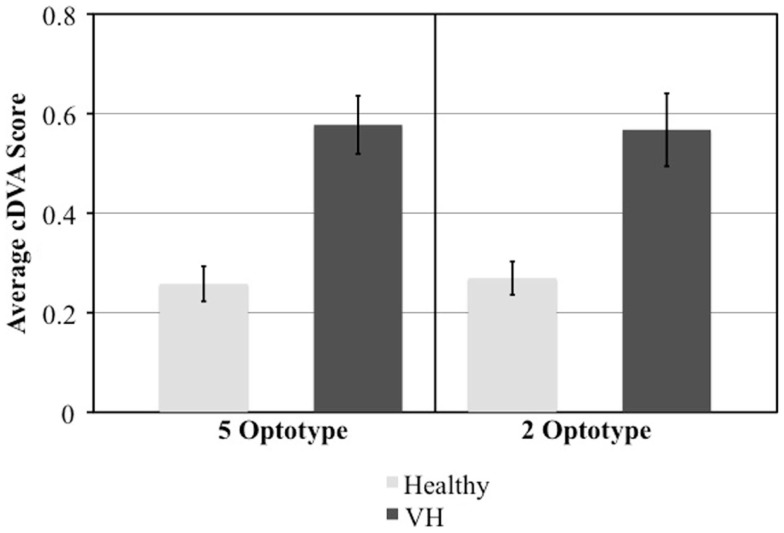
**Comparison of mean and standard error cDVA scores between those with and without vestibular hypofunction (VH) in the short (2 optotypes) and long (5 optotypes) version of the test**.

There was no difference between the short-cDVA test, displaying two letters per optotype size, and the long-cDVA test, displaying five letters per optotype size (*p* = 0.993). The agreement between the short-cDVA test and the previously validated long-cDVA test was excellent (ICC = 0.857, *p* < 0.001). Scores achieved by those with and without VH were significantly different on both long and short versions of the cDVA test (*p* = 0.0002 and *p* = 0.0025, respectively) (Figure 2). We examined sensitivity, specificity, and predictive value for individuals with vestibular deficits compared with healthy individuals using a criterion based on age-matched mean DVA score in the normative population +2SD. Results were comparable between the long- and short-cDVA tests, respectively (sensitivity 50 vs. 58%, specificity 100 vs. 100%, PPV 100 vs. 100%, NPV 63 vs. 67%).

**Figure 3:**
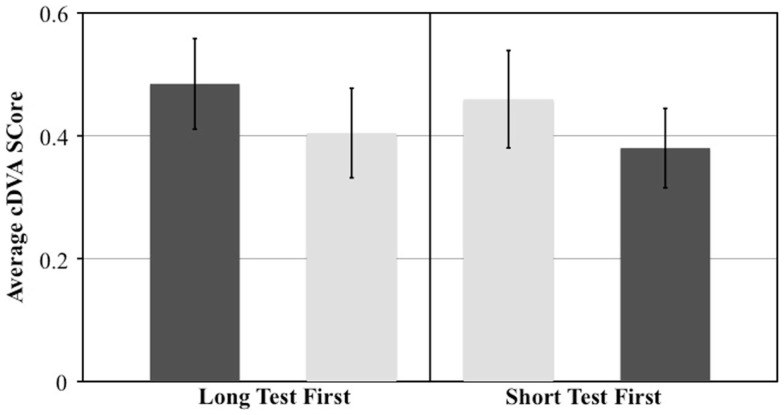
**Comparison of mean and standard error DVA scores based on test order**.

The effect of test order was evaluated by comparing the 11 individuals who underwent the long-cDVA test first with the 11 individuals who underwent the short-cDVA test first. We calculated the difference in DVA scores between the two tests and examined the effect of test order with a *t*-test. We observed a significant effect of test order on the difference in scores (*p* = 0.001). When the long-cDVA test was administered first, individuals scored 0.08 logMAR higher (worse) compared to the short-cDVA test. When the test order was reversed and the short-cDVA test was administered first, individuals scored 0.08 logMAR higher compared to the long-cDVA test (Figure 3). Regardless of test version, the DVA score obtained on the first test was found to be worse compared to the DVA score obtained on the second test.

## Discussion

The NIH Toolbox cDVA test is an easy-to-administer, valid, and reliable measure of peripheral vestibular function. In this study, we report normative scores in a large and diverse population of 3,992 individuals, aged 3–85 years. These scores can be utilized in conjunction with the NIH Toolbox test battery, available at www.nihtoolbox.org (11), in a variety of clinical and academic settings to measure outcomes in longitudinal epidemiologic studies and trials.

The effect of age and other sociodemographic characteristics on DVA scores was examined. In multiple regression analysis, we found an age-related decline in DVA. These results are corroborated by smaller studies that report age-related effects on DVA (4, 12, 13) and VOR function, particularly for higher velocities of head rotation (12, 14–18). In patients age 18–89, Paige et al. demonstrated age-related changes in VOR phase (timing relationship between head and eye velocity) and declining VOR responses during high-amplitude and high-velocity sinusoidal rotations (19). A 5-year longitudinal study of vestibular function observed a significant decrease in VOR gain to sinusoidal stimuli, specific to higher velocities (16). Our findings are also supported by histopathologic reports showing significant age-related declines in vestibular sensory hair cell populations in human temporal bones (20–22) as well as age-related neuronal loss in the human vestibular nucleus complex (23).

In this study, we demonstrated that DVA remains stable in individuals aged 3–49 years and then starts to decline at age 50 years. It has been shown that specific vestibular structures differentially degenerate with age. While sensory hair cell counts decrease by 6% per decade starting from birth (21, 24, 25), primary vestibular afferent fibers tend to degenerate from middle age on, with 35% of afferents remaining in individuals age 70–85 years (24, 26). Further histopathologic studies show that cells in Scarpa’s ganglion decline starting at age 30 with a steep decrease after age 60 (25, 27, 28), while vestibular nuclei neurons decrease by 3% per decade between 40 and 90 years of age (23, 29). It has been demonstrated that increased sensitivities of afferent nerve fibers and central mechanisms can compensate for earlier-onset hair cell loss, thus, maintaining normal function, with degeneration becoming more evident at older ages, beginning at midlife (30). Therefore, our observation that DVA remains stable until middle age, after which scores tend to decline, is supported by these differential age-related changes in various vestibular sensory components and suggests that DVA decline is specifically related to reductions in afferent and/or central vestibular function that start in midlife.

We found that cDVA scores were higher (worse) in males compared to females on adjusted analyses. Existing evidence surrounding gender-related functional or anatomic differences in the vestibular system is scarce. Three-dimensional measurements of the human vestibular apparatus have shown that males tend to have larger diameter semicircular canals (31), although these anatomic differences have not been directly correlated with vestibular testing results. One other epidemiological study of vestibular function in over 6,000 participants from the national health and nutrition examination survey (NHANES) evaluated balance function using modified Romberg testing and demonstrated no effect of gender on the prevalence of vestibular dysfunction (32). However, the postural tests used in this NHANES study can also reflect other sensory inputs, central processes, and motor ability, which may also be influenced by cardiovascular risk factors, while the DVA test targets VOR function. It is possible that the gender differences we describe are related to recent evidence suggesting that women are better able to discriminate between colors (i.e., flashing black optotype against a white background), putatively owed to gender differences in androgen receptors within visual cortex (33). Future studies are needed to more firmly establish any sex differences in vestibular function.

To administer the NIH Toolbox DVA test in a large, normative population, the number of letters administered per line (optotype size) was reduced from five to two and an ancillary study was conducted to compare the long and short versions of the test. We demonstrated an excellent agreement between long and short versions of the test. Additionally, DVA scores between individuals with and without VH were significantly different on both tests. Both tests exhibited excellent specificity and PPVs but poor to fair sensitivity and NPVs. It is important to note, however, that this ancillary study examined a small number of individuals and that 50% of those with vestibular pathology suffered from unilateral conditions over varying time courses. Therefore, it is possible that some individuals with vestibular pathology had compensated, likely from contralesional VOR gain restoration or reduced latency of compensatory saccades, and thus, exhibited improved gaze stability during the testing session (34). Further investigations with larger populations with more detailed classifications of vestibular pathology are warranted. However, given the overall results, both test versions are shown to be easily administered, reliable, and valid measures of DVA.

Although a short-practice trial was administered prior to data collection, we observed a significant learning effect in which performance improved on the second test, regardless of which version (long vs. short) was administered first. Though not explicitly the same type of DVA testing, these findings are consistent with previous studies documenting training effects in participants’ ability to resolve target motion, with wash-out periods ranging from 7 to 36 days (35–37). The learning or training effect should be acknowledged in future studies utilizing this behavioral measure of VOR function and more extensive practice trials may be considered to better familiarize subjects before data collection in future studies.

Several limitations of this study should be noted. Because this was a cross-sectional study, causal inferences cannot be made. Although the norming study was conducted in a diverse population from various regions of the United States, the smaller ancillary study evaluating post-validation changes to the test did not include children or Spanish speakers, thus, corresponding findings may not be generalizable to the entire population. Potential methodological considerations of DVA testing should also be considered. Self-generated head movements are associated with enhancements in gaze stability that may be due to an efference copy that can enable motor adaptation in successive eye movements (38) and/or centrally preprogramed eye rotations that increase VOR gain, defined generally as eye velocity/head velocity (39–45). Thus, passive DVA testing paradigms utilizing transient, unpredictable head thrusts in the plane of the semicircular canal of interest may better isolate and quantify peripheral vestibular function compared to active head rotation paradigms, such as the one used in this study. However, to comprehensively assess an individual’s gaze stabilization mechanisms during head movements that reflect daily activities, DVA testing utilizing active or voluntary head movements may be more appropriate as both VOR functionality and non-VOR gaze stabilization mechanisms may be recruited (46).

## Conclusion

We report normative scores for the previously validated NIH Toolbox cDVA test in a diverse population composed of nearly 4,000 individuals, aged 3–85 years. These data agree with the body of literature showing age-related changes in vestibular function and explores the effect of other sociodemographic characteristics, including gender, ethnicity, education level, and primary language. Findings from this study may aid in the interpretation of DVA scores across a diverse population and serve as a reference for future epidemiological investigations using the NIH Toolbox DVA test.

## Conflict of Interest Statement

The authors declare that the research was conducted in the absence of any commercial or financial relationships that could be construed as a potential conflict of interest.
